# Normalized, Segmented or Called aCGH Data?

**Published:** 2007-09-17

**Authors:** Wessel N. van Wieringen, Mark A. van de Wiel, Bauke Ylstra

**Affiliations:** 1 Department of Mathematics, Vrije Universiteit De Boelelaan 1081a, 1081 HV Amsterdam, The Netherlands; 2 VU University Medical Center, P.O. Box 7075, 1007 MB Amsterdam, The Netherlands

**Keywords:** array CGH, data, pre-processing, normalization, segmentation, calling

## Abstract

Array comparative genomic hybridization (aCGH) is a high-throughput lab technique to measure genome-wide chromosomal copy numbers. Data from aCGH experiments require extensive pre-processing, which consists of three steps: normalization, segmentation and calling. Each of these pre-processing steps yields a different data set: normalized data, segmented data, and called data. Publications using aCGH base their findings on data from all stages of the pre-processing. Hence, there is no consensus on which should be used for further down-stream analysis. This consensus is however important for correct reporting of findings, and comparison of results from different studies. We discuss several issues that should be taken into account when deciding on which data are to be used. We express the believe that called data are best used, but would welcome opposing views.

## Introduction

Chromosomal aberrations are a key event in the development and progression of cancer (Lengauer et al. 1998). Array comparative genomic hybridization (aCGH) is a high resolution method to detect these DNA copy numbers (Pinkel and Albertson, 2005). The increasing number of publications using aCGH (confer *arrayCGHtracker* at http://www.progenetix.de/) is based on data from different stages of the pre-processing. The pre-processing consists of three steps: normalization, segmentation and calling (discussed in detail in the next section). Here we argue that calling should be the last pre-processing step of aCGH data, and its resulting data are best used for down-stream analysis. We first introduce aCGH and its pre-processing steps. Then we discuss several issues that should be taken into account when deciding on which data are to be used.

## aCGH and pre-processing

Chromosomal DNA copy number is the number of copies of genomic DNA. Normal somatic cells have two copies of the autosomal chromosomes: the copy number is two. In addition, cells of normal males have one copy of the X and of the Y chromosome. Nuclei of Down syndrome patients show an extra copy of chromosome 21, whereas in cancer tissue the copy number may vary considerably over the genome.

Solinas-Toldo et al. (1997); Pinkel et al. (1998); Pollack et al. (1999); and Snijders et al. (2001) showed that DNA copy number can be measured genome-wide using aCGH. aCGH is a high-throughput technique, similar to the gene expression microarray but uses chromosomal DNA rather than cDNA for the hybridization. The array consists of many (possibly synthetic) strands of DNA, here referred to as array elements, that interrogate small regions of the genomic DNA (Pinkel and Albertson, 2005).

In an aCGH experiment is differentially labeled test and reference samples are hybridized together to the array. The reference sample is assumed to have copy number two for all somatic chromosomes. Image analysis then results in test and reference intensities for all array elements. Under ideal experimental conditions, the intensity of an array element is linearly proportional to the abundance of the corresponding DNA sequence in the sample. The log_2_ ratio of the test and reference intensities reflect the relative copy number in the test sample compared to that in the reference sample.

Pre-processing comprises of all preliminary operations on the data necessary to arrive at the quantity of interest. As such it is, together with the experimental protocol, an inherent part of the operational definition of the property being measured, the copy number of a genomic segment. For aCGH, the log_2_ ratios undergo three preprocessing steps before arriving at the actual copy number.

- The first pre-processing step is normalization (Khojasteh et al. 2005; Neuvial et al. 2006). Normalization corrects for experimental artifacts in order to make the log_2_ ratio’s from different hybridizations comparable.
- The second step of the pre-processing, named segmentation, is motivated by the underlying discrete DNA copy numbers of test and reference samples. Segmentation algorithms divide the genome into non-overlapping segments that are separated by breakpoints (Jong et al. 2004; Olshen et al. 2004; Fridlyand et al. 2004; Picard et al. 2005; Marioni et al. 2006). These breakpoints indicate a change in DNA copy number. Array elements that belong to the same segment are assumed, as they are not separated by a breakpoint, to have the same underlying chromosomal copy number. Segmentation methods also estimate the mean log_2_ ratio per segments, referred to as states.
- As a final and last pre-processing step, referred to as calling (Wang et al. 2004; Broët and Richardson, 2006; Engler et al. 2006; Van de Wiel et al. 2007), the DNA copy number of each segment is determined. At present calling algorithms cannot determine whether there are, say, three or four copies present. They can however detect deviations from the normal copy number, and classify each segment as either ‘normal’, ‘loss’, ‘gain’ or ‘amplification’: ‘normal’ if there are two copies of the chromosomal segment present, ‘loss’ (also named deletion) if at least one copy is lost, ‘gain’ if at least one additional copy is present, and ‘amplification’ if there are high level, say >5, copy numbers (for simplicity we ignore the existence of polyploid genomes). These labels are referred to as calls.

Pre-processing thus maps the raw log_2_ intensity values onto the ordinal scale of the calls.

**Figure 1.**
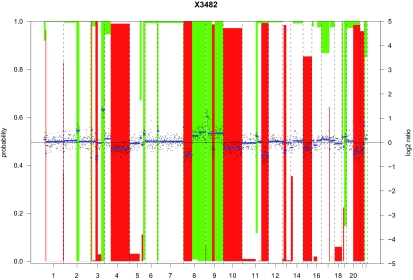
Pre-processing results of the oral squamous cell carcinoma sample X3482 from the data set of Snijders et al. (2005).

Figure 1 shows the different pre-processing data for an oral squamous cell carcinoma sample from the data set of Snijders et al. (2005). The data have been median normalized, segmented with DNAcopy of Olshen et al. (2004) and called with CGHcall of Van de Wiel et al. (2007). Normalized log_2_ ratios are plotted (black dots) with the scale on the right axis. The segmentation states are plotted in blue. Red and green bars indicate loss and gain probabilities, respectively. The left axis contains the probability scale, which is reversed (“1–”) for the gains. Segments for which either bar extends beyond the middle axis (probability >0.5) are aberrated.

Table 1 contains the pre-processed data of the same oral squamous cell carcinoma sample. It should be observed that the normalized data vary between clones, whereas the segmented and called data only exhibit a change at the breakpoints found by DNAcopy.

Each step of the data pre-processing yields a different data set: normalized data, segmented data, and called data. There is no consensus on which should be used for further down-stream analysis. For instance, Weiss et al. (2003) and Ching et al. (2005) use normalized data, Jong et al. (2007) and Fridlyand et al. (2006) use segmented data, and Jönsson et al. (2005) and Zhao et al. (2005) use called data. This consensus is important for correct reporting of findings, and comparison of results from different studies.

## Discussion

Several aspects of the different data and issues surrounding their use in analysis of aCGH experiments are discussed. This should help statisticians and bioinformaticians in their decision on the data to be used.

### Interpretation

The called data have a clear biological meaning, lacked by the normalized or segmented data. The ramifications—with respect to copy number —of a statement like ‘8q3 has a loss’ are evident: at most one copy of 8q3 is present. The ramifications of ‘8q3 has a log_2_ ratio of −0.17’, however, are less clear. Similarly, how is the difference between two segmentation states to be interpreted in terms of their difference in copy number?

### Platform comparison

aCGH profiles from different platforms can be compared directly when using the called data: the interpretation of ‘loss’, ’normal’, ‘gain’ and ‘amplification’ is the same across platforms, whereas the segmentation states are likely to have different interpretations between platforms, possibly even between experiments. The calling could thus be viewed as a final between-array normalization step. This comparability is a requirement for down-stream analysis, which involves data from multiple hybridizations. A requirement satisfied by the called data, but not ensured for the segmentation states.

### Corroboration

The called data can directly be used for corroboration with existing knowledge of genomic aberrations, which is expressed in terms of copy numbers. This corroboration may function as an internal control of the experiment. For instance, called data from a human male sample show a loss at the X chromosome. Of course, the normalized or segmented data could be used for the same purpose: a plot will reveal a jump in the data. Nonetheless, one still needs to decide whether this jump corresponds to an aberration. Most likely, one will (implicitly) draw an imaginary line used as a yardstick for classification, thus mapping the normalized or segmented data to the ordinal scale of the called data.

### Verification of findings

The verification of findings from an aCGH experiment is often done by FISH (e.g. Snijders et al. 2001; Van den IJssel et al. 2005). FISH counts the copy number of a genomic segment of interest. Hence, not the log_2_ ratio is verified, but the call of (say) a loss. The latter has tangible implications for the copy number that can be verified (or falsified) using FISH: is there a reduced copy number? Hence, the scale of the calls directly matches that of the outcome of verification experiments, unlike the normalized and segmented data which need to be translated to the scale of aberrations, the scale on which new findings are reported.

### ‘De-noising’

The DNA copy number assumes only integer values, {0, 1, 2, 3,...}. The discreteness of this scale pleas for a discretization of the normalized data, which could be viewed as ‘de-noising’ the normalized data. Both segmented and called data are ‘de-noised’ data. Willenbrock and Fridlyand (2005) showed that dealing with aCGH data as discrete levels (segmentation states) rather than normalized log_2_ ratios greatly improved sensitivity and specificity.

### Cell heterogeneity

aCGH detects chromosomal aberrations if they are present in the majority of the cells of the test sample. Heterogeneity in the DNA copy number profiles between cells complicates the calling, possibly leading to incorrect calls. Similarly, it introduces differences in segmentation states and additional breakpoints that are not unambiguously assignable to changes in copy number. Where the heights of the segmentation states reflect—in some intricate way—the cell heterogeneity, the calls contain no information on the heterogeneity. This is straightforwardly overcome when not the actual calls, but their (posterior) probability of a ‘loss’, ‘normal’, and ‘gain’ (as provided by CGHmix of Broët and Richardson, 2006; or CGHclassify by Engler et al. 2006) are considered as the resulting data from a calling method. These probabilities reflect the cell heterogeneity: the probability of a loss corresponds to the proportion of cells with a loss in the sample.

“On the other hand, in cases of tumors with instable karyotypes, the fact that random gains and losses of chromosome material affecting only a few cells cannot be ascertained should help tremendously in distinguishing chromosomal imbalances present in the majority of cells of a given tumor. (a)CGH analyses performed with tumor DNA prepared from a series of individual tumors representing a distinct tumor type should lead to the identification of those chromosomal imbalancies that are consistently involved, and should thus help identify candidate chromosome segments for genes of major biological importance for the tumor type in question.” (Du Manoir et al. 1993).

Not the heterogeneity of a tumor, but knowledge on the presence or absence of chromosomal aberrations in a tumor is what the researcher is generally after when profiling a tumor by aCGH. If one is interested whether these aberrations occur in all or part of the tumor one would need to perform targeted experiments by taking separate samples of different parts of a tumor.

### Imperfect calling

The calling of aberrations is not yet perfect in case samples or chromosomal areas contain different log-ratio levels for the same copy number. For example, due to unknown contaminations by normal cells or different ploidies. In principle, this ‘standardization imperfection’ is also present in the normalized and segmented data, but has less consequences due to the continuous levels (a sample with low gain log_2_ ratio levels still contributes to the total signal).

The problem of imperfect calling may be resolved when the call probabilities (instead of the calls) are used in down-stream analysis, for call probabilities reflect the uncertainty of the calling. Then, like low gain log_2_ ratio levels, small call probabilities of a gain still contribute. As such the use of call probabilities in down-stream analysis could thus be viewed as error propagation.

Moreover, calling methods are likely to improve (the first paper on calling, Wang et al. 2004, is only a few years old) and address these matters.

### Down-stream analysis

Before we discuss the use of called aCGH data in several instances of down-stream analysis, it should be noted that most machinery for the analysis of continuous expression data is not applicable to the ordinal called aCGH data. Tailormade methods for the analysis of called aCGH data are needed. This can only be considered as a challenge for the statistical community, and only a temporary, practical, not an intrinsic, argument in favor of the use of normalized or segmented data in down-stream analysis.

### Hypothesis testing

The use of called data may lead to a loss of statistical power when testing hypotheses, if the calling discretizes the copy numbers too much. This lack of power may be overcome by the use of call probabilities in hypothesis testing, or as described below (see *Hypothesis testing revisited*). An approach taken (e.g. Nakao et al. 2004) to circumvent this possible loss of statistical power is the use of the normalized data. But what conclusion can one draw if the null hypothesis *H*_0_ is rejected using the normalized data? Only that the average signals differ, NOT that the copy number differ. Hence, inferences with respect to copy number differences on the basis of normalized (or segmented) data are formally invalid. The called data facilitate inferences on the desired biological level, that of the copy numbers.

### Dimension reduction

The called data allow for natural dimension reduction of the data (Van de Wiel and Van Wieringen, 2007). This is done by changing the statistical unit from array elements into regions. A region is a series of neighboring array elements on the chromosome whose aCGH-signature is shared by all array elements. The ordinal nature of the aCGH data allows for a huge dimension reduction, as the data assume only a limited number of values (three or four), many segments on the genome are likely to have the same values over consecutive array elements. A region may consist of one array element representing a small amplification, but also a complete chromosome arm. Hence, the dimension reduction from array elements to regions captures the relevant features of the data.

Such a natural, interpretation preserving reduction of the data seems not possible with normalized data. Of course, a technique like principal component analysis could be applied. This would yield principal components that are weighted averages of the aCGH profiles. These components could be interpreted, following Alter et al. (2000), as ‘superclones’. Such an interpretation is however a label without content, for it is neither linked to a biological entity nor to a theoretical construct. The interpretation of components is generally not straightforward, especially if the number of array elements that contribute to the component gets large. Nonetheless, these components can be used for low-dimensional visualization. But such components can also be constructed for the called data, as is outlined in Jöreskog and Moustaki (2001).

### Hypothesis testing revisited

The use of dimension reduced regional called data can be very advantageous for down-stream analysis (Van de Wiel and Van Wieringen, 2007), as a re-analysis of the colorectal cancer data set of Douglas et al. (2004) shows. The original analysis of the data set reported several interesting findings, but lacked rigorous statistical evidence (for instance, *p*-values were not mentioned). In Van de Wiel and Van Wieringen (2007) the data were re-analyzed applying CGHMultiArray (Van de Wiel et al. 2005), a tailor-made two-group testing procedure for ordinal data, to the regional called data. This confirmed most of the findings reported by Douglas et al. (2004), now provided with a sound statistical underpinning. Recall that, for reasons discussed above (see *Hypothesis testing*), copy number differences between the two conditions could not have been inferred (formally) from the normalized or segmented data.

### Unsupervised analysis

Finally, we discuss the use of aCGH data for unsupervised analysis (omitting supervised analysis of aCGH as it has not received much attention yet). Normalized or segmented data are often used for clustering of aCGH data (e.g. Wilhelm et al. 2002; Jong et al. 2007). Effectively, this means the signal—as is, regardless of its biological interpretation—is used as input, hoping for sensible output. The unsupervised method is then treated as a black box. Its results may be very interesting. But they may also be hard to explain in terms of what aCGH purports to measure, DNA copy number.

The use of the called data in unsupervised analysis can be advantageous (Van Wieringen et al. 2007). The use of regions, rather than array elements, as input of the cluster method introduces a natural and data-driven weighting in the clustering. Long ‘dull’ chromosomal areas with normal DNA copy number and small amplifications are weighted equally. One thus clusters on the relevant features of the data, without letting dull areas dominate the cluster output. Simulations in Van Wieringen et al. (2007) showed that the use of regional called data improves the performance of clustering methods, even outperforming clustering methods using the normalized data.

## Conclusion

The discussion above reveals that called data have the clearest interpretation, are directly translatable to tangible implications for the empirical relationships between DNA copy numbers (whereas normalized and segmented data are not), are a necessity for inferences on the DNA copy number, and can be advantageous for down-stream analysis.

The resolution of calling methods could be improved though, for instance calling into the classes ‘double deletion’, ‘single deletion’, ‘normal’, ‘single copy gain’, ‘double copy gain’ and ‘amplification’. Furthermore, heterogeneity of the cell population suggests that the distribution of the calls may be preferred. For data from a homogeneous cell population, however, the actual calls suffice, as the probability will be close to one. Also tailor-made methods for the analysis of called aCGH data are needed.

In all, calling is an inherent part of the pre-processing, yielding the quantity most closely linked with copy number. Realizing that improvement and development of calling methods and down-stream analysis methods are needed, we currently believe that it is this quantity, the call or call probability, that is to be used.

We do, however, not claim that the above discussion is exhaustive and final. For instance, it is not known yet which data are best used for prediction purposes. We would therefore welcome additional arguments and opposing views on the issue, and hope that a thorough discussion will yield the desirable consensus on which pre-processed aCGH data are to be used.

## Figures and Tables

**Table 1.** Normalized log_2_ ratio’s, segmentation states, and calls for a small part of the genome of the oral squamous cell carcinoma sample X3482 from the data set of Snijders et al. (2005). The coding of the calls is as follows: −1, 0, 1 correspond to loss, normal, gain, respectively.

**Clone ID**	**Chr.**	**Start bp.**	**Normalized log**_**2**_**ratio**	**Segmentation state**	**Call**
⋮	⋮	⋮	⋮	⋮	⋮
RP11–232D21	4	48102	0.239	0.151	0
RP11–109P3	4	48340	0.291	0.151	0
RP11–72P14	4	48340	0.380	0.151	0
RP11–217B22	4	48819	0.227	0.151	0
RP11–32K21	4	52945	–0.348	−0.250	−1
RP11–210D19	4	53294	–0.216	−0.250	−1
RP11–175I24	4	53657	–0.319	−0.250	−1
RP11–98G22	4	54688	0.000	−0.250	−1
⋮	⋮	⋮	⋮	⋮	⋮
